# Ultra-Processed Food Consumption and Irritable Bowel Syndrome: Current Evidence and Clinical Implications

**DOI:** 10.3390/nu17223567

**Published:** 2025-11-14

**Authors:** Hanna Fjeldheim Dale, Marit Kolby, Jørgen Valeur

**Affiliations:** 1 Unger-Vetlesen Institute, Lovisenberg Diaconal Hospital, 0456 Oslo, Norway; jorgen.valeur@lds.no; 2 Department of Health, Oslo New University College, 0454 Oslo, Norway; marit.kolby@oslonh.no; 3 Institute of Clinical Medicine, University of Oslo, 0372 Oslo, Norway

**Keywords:** irritable bowel syndrome, brain–gut axis, disorder of gut–brain interaction, ultra-processed foods, microbiome

## Abstract

Irritable bowel syndrome (IBS) is a prevalent disorder of gut–brain interaction (DGBI) with an adverse impact on quality of life. The global consumption of ultra-processed foods (UPF) is rapidly increasing, and UPF intake has recently been linked to a wide range of metabolic and chronic diseases. The potential role of UPF consumption in the onset and symptom generation of IBS is emerging but remains unclear. This narrative review synthesizes epidemiological evidence on the association between UPF consumption and IBS, integrates mechanistic insights from experimental and clinical studies and suggests clinical implications based on the current state of knowledge. Observational studies suggest that higher UPF intake may be associated with increased risk of IBS, although the evidence base is limited and subject to methodological challenges. Mechanistic studies indicate that additives including emulsifiers and non-nutritive sweeteners can alter pathways relevant to IBS symptom generation, such as gut microbiota composition, impair intestinal barrier function and trigger low-grade inflammation. Current evidence supports a possible link between UPF consumption and IBS. Increasing overall dietary quality and reducing UPF intake are promising complementary strategies to established dietary interventions. Future intervention trials may provide insights into relevant biological mechanisms, particularly if such changes co-occur with symptom improvement.

## 1. Introduction

Irritable bowel syndrome (IBS) is one of the most prevalent disorders of gut–brain interaction (DGBI), affecting a substantial part of the population worldwide and leading to reduced quality of life [1]. IBS is defined by Rome IV criteria as a condition of abdominal pain related to disordered bowel habits, associated with a wide range of both gastrointestinal (GI) and extraintestinal symptoms [2]. Despite decades of research on IBS pathophysiology, root causes are largely unknown, and consequently the treatment to date is mostly targeting symptoms [3]. Notably, the concern about the role of diet in IBS development is constantly increasing. Despite the well-known role of diet in IBS symptoms, the specific impact of food processing has been underexplored until recently. Accumulating evidence indicates that dietary triggers are important aggravators on IBS symptoms [4,5,6], and along with the general concern about negative health outcomes from consuming ultra-processed foods (UPF), recent results from epidemiological studies show associations between intake of UPF and increased risk of gut disease, including IBS [7,8].

Hence, now more than ever, it is essential to identify key modifiable dietary risk factors to reduce the burden of IBS. The aim of this narrative review is to highlight existing evidence on the association between IBS and the intake of UPF, integrate mechanistic hypotheses and suggest clinical implications based on current state of knowledge.

## 2. Methods

The literature relevant to this narrative review was identified through PubMed using adapted variations of keywords relating to ultra-processing, such as «ultra-processed foods», «additives», “non-nutritive sweeteners”, «emulsifiers», and relevant outcomes, such as «irritable bowel syndrome», «inflammatory bowel disease», «microbiota», «microbiome» and «brain–gut axis». The main search term was “ultra-processed foods” OR “emulsifiers” AND “irritable bowel syndrome.” The collection of data was performed between 11 August and 23 September 2025 and included in vitro and in vivo models, animal models, epidemiological studies and human clinical trials. No evidence grading or quality assessment was performed. Only articles published in English were considered. In the subsequent sections, results are presented and discussed continuously.

## 3. Definition and Classification of Ultra-Processed Foods

Defining foods by their level of industrial processing has been subject to much debate, as at least eight different classification systems have historically been used for this purpose [7]. The differences between definition systems make comparison between studies that use different process-classifications challenging. The definition of UPF remains debated, with some researchers arguing that the classification overly emphasizes ingredient lists rather than specific processing techniques or nutritional characteristics [9]. Notably, the NOVA-classification is the most widely used classification system, adapted by the Food and Agriculture Organization of the United Nations [10].

The NOVA-classification divides foods into four different groups depending on both the degree and purpose of industrial processing they have undergone. UPF are described as “evolutionarily novel, industrial formulations manufactured by deconstructing foods into their component parts (such as oils, starches and sweeteners), modifying them (for example, through enzymatic processes) and recombining them with cosmetic additives” [11]. It includes ready-to-consume products such as sweets, snacks, pastries, breakfast cereals, ready meals, meat, meat products and meat replacements, ice-cream, energy bars, instant sauces and soups and cocoa, fruit and milk drinks [12]. UPFs are typically energy-dense products with poor nutritional profiles, seen as products high in sugars, fat and salt and low in dietary fibres, vitamins and minerals. In addition, UPFs contain a broad range of non-natural ingredients and additives. These foods tend to displace foods with better nutritional value and give less feeling of satiety than minimally processed foods, hence causing a higher glycemic response and increasing the risk of consuming excess energy [13].

UPF products have been aggressively marketed globally in recent decades, given the hyperpalatable and convenient nature of most UPF products (durable, ready to consume). Data show that UPF comprise more than 50% of total daily energy intake in high-income countries such as USA and Canada, with considerably higher intake in young people [14,15,16,17]. It must be noted that factors associated with higher UPF intake are, among others, reported to include lower income and educational level, living alone, overweight and obesity, low level of physical activity and greater screentime during meals [7]. These factors might be insufficiently adjusted for in epidemiological studies, which can be regarded as a limitation to results from studies on UPF.

## 4. Diagnosis, Symptoms, Prevalence and Comorbidities

IBS is characterized by fluctuating abdominal pain and altered bowel habits according to the Rome IV criteria, and depending on the predominant bowel habit it is classified as IBS-diarrhea (IBS-D), IBS-constipation (IBS-C), IBS-mixed (IBS-M) or IBS-unsubtyped (IBS-U) [18]. The estimated prevalence of IBS is between 5 and 10% in most parts of the world, with the caveat of missing data from the Middle East, Eastern Europe and Africa [18].

In addition to GI symptoms, IBS is commonly accompanied by comorbidities such as depression, anxiety, sleep disturbances, chronic pain and fatigue [3]. In a Norwegian online survey from 2025, including 2400 IBS patients, as many as 97% of patients stated that IBS-symptoms reduced their quality of life [19]. IBS poses a high cost for society as it affects a huge proportion of the population and reduces the work capacity and increases the health costs of those affected [20]. Given the high burden IBS poses both on society and on patients, it is imperative to develop both preventive and therapeutic measures to improve IBS outcomes.

## 5. IBS Pathophysiology—A Complex Picture

Despite decades of targeted research, the exact pathophysiology of IBS remains unclear. Current knowledge points towards a dysfunctional interplay between the gut and the central nervous system as a key feature, seen as a disruption in the gut–brain axis [21]. Several pathways are suggested to generate symptoms in IBS, such as gut microbiota alterations, low grade inflammation, impaired intestinal barrier function, altered motility and visceral hypersensitivity [3,22]. Lately, IBS as a solely functional disorder has been questioned, as subjects with IBS exhibit both gut-specific and systemic signs of inflammation-related changes, compared to healthy controls [3,23,24]. Higher levels of anxiety and depression are reported in IBS patients than in controls [25]. Such comorbidities may both influence and be influenced by diet, suggesting that this interplay is of importance in the IBS pathophysiology.

The gut microbiota profile of IBS patients is reported to differ from healthy controls [26,27,28,29]. Although a single ‘universal’ beneficial gut microbiota composition cannot be defined, gut health depends more on microbial functions, ecological balance and host context than on any fixed taxonomic profile. Still, increasing evidence from the last decade point towards gut microbiota disturbances as a hallmark of a wide range of disorders, including IBS [30,31]. Given this knowledge, improving gut microbiome function by optimalisation of diet could be an effective strategy to target one of the known drivers of IBS pathophysiology.

## 6. Dietary Management and Dietary Patterns in IBS

Food intake is a well-known symptom trigger for those suffering from IBS, and between 62% and 90% of all patients report worsening of symptoms related to intake of specific foods [32]. This could be explained by atypical modulatory mechanisms in the gut in response to the stimulation mediated by food intake, as nutrients present in the GI tract affect GI motility, barrier function, visceral sensitivity and gut microbiota composition [33]. Hypersensitivity to certain foods is shown to cause low-grade intestinal inflammation and increase visceral sensitivity and intestinal permeability [34]. Many patients adopt a self-directed exclusion diet, and differences in habitual dietary intake and poorer overall dietary quality have been observed in people with IBS compared with controls [35]. Of note, 1/3 of all patients do not experience sufficient symptom relief when following evidence-based dietary guidelines [1,36].

Dietary management, including restriction of FODMAPs (fermentable oligo-, di-, mono-saccharides and polyols), has in recent decades been an important tool in the management of IBS symptoms. Evidence from randomized trials and network meta-analyses indicates that the low-FODMAP diet produces the largest and most consistent short-term symptom relief [37,38]. Of note, increasing evidence shows that Mediterranean-style diets appear feasible and can improve gastrointestinal and psychological symptoms in IBS [39,40]. Also, a very low-carbohydrate diet has been found to improve symptoms and quality of life in IBS-D [41]. Emerging population studies also show that certain dietary patterns, including high consumption of ultra-processed foods, “fast food” and ‘Western’ dietary patterns, are associated with higher prevalence or incidence of IBS and other disorders of gut–brain interaction, whereas vegetarian and Mediterranean dietary patterns are associated with reduced risk of IBS [42,43].

Dietary patterns exert a robust influence on the composition and function of the human gut microbiome, which in turn modulates mucosal immune homeostasis by shaping the balance between pro- and anti-inflammatory responses. Dietary patterns high in processed and non-fish animal foods are associated with higher abundances of Firmicutes-dominated taxa and endotoxin-synthesis pathways, whereas patterns rich in whole plant foods and fish are positively associated with short-chain fatty acid-producing commensals [44]. As the microbiome is a central component in IBS pathophysiology, the dietary pattern is supposedly decisive for both IBS risk and symptom generation.

## 7. Epidemiological Evidence Linking UPF to IBS

An increasing number of cohort studies and meta-analyses of cohort studies have reported associations between higher intake of UPF and mortality [45] and morbidity, in particular greater risk of coronary heart disease, cardiovascular disease, type 2 diabetes mellitus and cancer [8,46]. Also, accumulating evidence points towards UPF as a factor that increases the risk of disorders in the GI tract, including inflammatory bowel disease (IBD), disorders of gut–brain interaction (DGBIs) and intestinal cancer [7]. Evidence on the association between UPF and IBS is limited; however, novel research from recent years points towards high intake of UPF as a risk factor of IBS (Table 1) [47,48,49].

The cross-sectional French NutriNet-Santé cohort study investigated the association between UPF intake and IBS in 33,343 subjects [48]. Dietary evaluation was based on at least three different 24 h food records. UPF consumption was defined according to the NOVA food classification system as % UPF of total food weight. For the cohort in total, UPF intake accounted for 16% of the food consumed in weight, corresponding to 33% in total energy intake. IBS diagnosis was made after completion of the Rome III questionnaire, and IBS was found to be present in 10.5% of the included subjects. After adjusting for confounding factors, they found that an increase in UPF consumption was associated with a higher risk of IBS [48]. The authors highlight the need for future studies to understand the relative impact of nutritional composition and specific characteristics of UPF related to IBS [48].

A large-scale prospective cohort study from the UK, including Biobank data from 178,711 participants during an 11-year follow-up, recently examined the long-term risk of IBS associated with UPF consumption [47]. Dietary evaluation was performed according to a 24 h dietary recall. UPF consumption was defined according to the NOVA food classification system as grams per day. The study included subjects during the years 2009 to 2012, free of IBS, celiac disease, IBD and any cancer at baseline. The diagnosis of IBS during follow-up was ascertained via ICD-10 codes (K58) up to May 2022. They reported the mean UPF consumption to be 21% of the total energy intake, and 8% higher risk of IBS was associated with every 10% increment of UPF consumption. The highest quartile of UPF consumption was associated with a significantly increased risk of IBS compared to the lowest quartile [47]. These results provide evidence for a dose–response relationship between intake of UPF and risk of IBS, and the authors highlight that their findings suggest that avoiding or lowering UPF consumption could be considered as a potential strategy to help address the increasing burden of IBS [47].

The cross-sectional Iranian ISFUN-study reported on the association between UPF intake and DGBIs, including IBS, in 1892 adults [49]. Dietary intake assessment was performed according to a validated 106-item semi-quantitative Food Frequency Questionnaire (FFQ), addressing both portion sizes and frequency of consumption. UPF consumption was defined according to the NOVA food classification system as grams per day. The prevalence of IBS was 5.3% according to Rome IV criteria [49]. Participants were categorized into tertiles of UPF energy contribution (% of kcal) (T1–3), and being in the highest versus the lowest tertile of UPF consumption was significantly associated with increased risk of IBS. The authors highlight that the contribution of UPFs to daily energy intake in their study population (around 9%) was substantially lower than in developed countries, and that the findings point towards the importance of emphasizing increased consumption of healthy whole foods and limiting the consumption of UPFs in IBS [49].

While the existing epidemiological studies consistently suggest a positive association between UPF intake and IBS risk, several methodological considerations must be acknowledged. All available studies rely on self-reported dietary data obtained through FFQs or repeated 24 h recalls, which are subject to recall bias and misclassification of UPF categories, especially given inconsistencies in NOVA application. Cross-sectional designs, such as the NutriNet-Santé and ISFUN studies, cannot exclude reverse causality, since individuals with gastrointestinal complaints may already avoid or increase UPF consumption in response to symptoms. The UK Biobank study provides the strongest evidence to date, owing to its large prospective design and long follow-up, yet residual confounding by unmeasured lifestyle or socioeconomic factors cannot be ruled out. In most cohorts, the strength of association was modest (odds or hazard ratios ranging from ~1.1 to 1.9 across exposure quartiles), comparable to other dietary risk factors. Importantly, several studies have reported that the associations persisted after adjustment for total energy intake, fibre and markers of overall diet quality, suggesting that factors intrinsic to processing itself may contribute to risk [7,47,48]. Nevertheless, the potential for residual confounding remains, and future prospective cohorts using objective biomarkers of dietary intake, repeated measurements and sensitivity analyses for socioeconomic and psychological covariates are warranted to strengthen causal inference.

Taken together, although novel results from epidemiological studies point out a clear association between UPF intake and risk of IBS, further experimental studies are needed to look at the mechanisms behind the effect of UPF intake in IBS and further investigate the effects of UPF on the GI tract. Current results can generate mechanistic hypotheses, but do not yet prove causality. Still, the findings to date suggest the reduction or elimination of UPF products as a dietary strategy to help mitigate the increasing burden of IBS, which is in line with suggested recommendations for the general population [50].

## 8. UPF and Diet: Thinking Beyond Nutrient Content

Although a broad proportion of products typically recognized as UPF can be classified as “fast food” or “junk food”, it has to be noted that a substantial part of food items that according to nutrient content typically would be considered as part of a healthy diet (such as yoghurts, whole meal bread, plant-based meat alternatives and most spreads and bread toppings), also are placed in the UPF category according to the content of additives. This means that—if dietary quality and nutrient intake is not adjusted for in a research setting—a person eating a lot of typical, unhealthy fast foods such as ready meals, pastries and take-away dinners can be put in the same UPF category as a person eating store-bought whole meal bread, fruit yoghurts and ready-made granola. If picking the presumably healthy UPF items, it is possible to follow national guidelines for a healthy diet in most Western countries, and until recently, there has been a lack of studies comparing healthy UPF diets to unprocessed diets.

Hall et al. have shown in a controlled feeding study in 20 healthy subjects that a high-UPF diet led to greater energy consumption and weight gain than an isocaloric unprocessed diet matched in macronutrients, whereas an “unprocessed” diet led to weight reduction, as well as improvements in appetite regulation hormones, in the absence of calorie restriction [51]. Novel findings by Dicken et al. from a randomized controlled trial, show that a UPF diet following healthy dietary guidelines lead to less weight loss than a matched diet of minimally processed foods [52]. Interestingly, the group eating a “healthy UPF-diet” reported more constipation and complaints typically associated with IBS than the group eating a minimally processed diet. The authors conclude that there is a need for dietary guidance on food processing, in addition to existing nutrient recommendations [52]. A novel dietary cross-over study in 43 healthy Danish men, investigated the effect of UPF consumption on male reproductive and metabolic health [53]. A comparison between three weeks on a UPF diet and three weeks on a minimally processed diet matched in nutrient content, clearly showed that the UPF diet led to reduced sperm quality and impaired cardiometabolic health [53].

In a recent publication from the Norwegian Women and Cancer cohort study, the authors chose to express UPF by weight rather than energy to be able to consider UPFs that do not provide any energy (such as artificially sweetened beverages and foods) and components that are added or created during processing, such as food additives and neo-formed components [54]. When UPF consumption was expressed per unit of energy rather than by weight, they observed no significant associations between UPF intake and colorectal cancer, but when UPF consumption was expressed by weight, the association was statistically significant [54]. This finding suggests that non-nutrient factors might be contributing to the negative health effects associated with high UPF intake, and that studies reporting UPF intake by energy might miss relevant artificially sweetened UPFs. Taken together, these findings contribute important new knowledge implicating that degree of processing affects our metabolism beyond the effect of nutrient content.

## 9. Potential Biological Mechanisms Linking UPF Consumption to IBS

**Figure 1:**
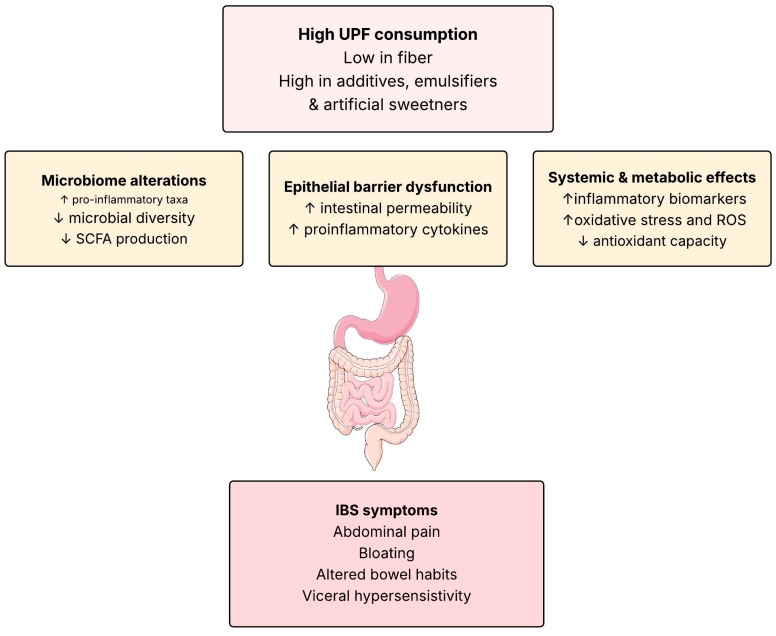
Conceptual model of potential mechanisms linking ultra-processed food consumption and IBS. High intake of UPFs may alter gut microbial composition, disrupt epithelial barrier function and trigger immune activation and low-grade inflammation. These intestinal changes, combined with oxidative stress and systemic metabolic disturbances, can influence gut–brain signalling and promote characteristic IBS symptoms such as abdominal pain, bloating and altered bowel habits. SCFS: Short chain fatty acids, ROS: Reactive oxygen species, ↑: increased, ↓: decreased. Illustrations by SMART Servier Medical Art.

The relationship between UPF consumption and IBS likely involves multiple, overlapping biological pathways. These can broadly be categorized into (a) microbiome-mediated processes, (b) inflammation and epithelial barrier dysfunction and (c) systemic and metabolic responses. A conceptual model of potential mechanisms linking ultra-processed food consumption and IBS is shown in Figure 1. Together, these mechanisms may contribute to the generation and maintenance of IBS symptoms through effects on gut–brain signalling, immune activation and visceral sensitivity.

### 9.1. Microbiome-Mediated Processes

UPFs are typically characterized by a low content of dietary fibre and resistant starch, and by the inclusion of food additives such as emulsifiers, artificial sweeteners and modified starches. This compositional profile may unfavourably alter the gut microbiota and its metabolic output. Results from a human feeding study have demonstrated that dietary emulsifiers, including carboxymethyl cellulose (CMC) and polysorbate-80 (P-80), can reduce microbial diversity, promote the expansion of pro-inflammatory taxa such as *Enterobacteriaceae* and displace commensal bacteria from the mucus layer [55]. This is supported by results from animal models suggesting CMC, P-80 and glycerol monolaurate to cause altered gut microbiota composition associated with increased pro-inflammatory potential in animals [56].

Maltodextrin—a common additive in UPFs—has also been shown to impair mucus production and reduce goblet-cell maturation, thereby facilitating bacterial adherence to the epithelium [57]. These microbial shifts may reduce short-chain fatty acid (SCFA) production, including butyrate, a key metabolite for epithelial energy supply and anti-inflammatory signalling. In human cohorts, high UPF intake has been associated with lower abundances of *Faecalibacterium prausnitzii* and *Roseburia* species and with altered fecal metabolomic profiles, indicating reduced SCFA synthesis [58,59]. Such changes could lead to reduced microbial resilience, increased mucosal permeability and amplification of local immune responses, all of which are relevant to IBS pathophysiology.

### 9.2. Inflammation and Epithelial Barrier Dysfunction

Another proposed mechanism is that UPF components directly impair epithelial integrity and activate mucosal immune pathways. An RCT in human subjects with ulcerative colitic (UC) in remission, have shown that the common additive carrageenan can induce inflammatory signalling through activation of the Toll-like receptor 4 (TLR4) and NF-κB pathways, leading to secretion of pro-inflammatory cytokines (IL-6, IL-8, TNF-α) and disruption of tight junction proteins [60].

Animal studies confirm that carrageenan exposure increases intestinal permeability, facilitates bacterial translocation and aggravates experimental colitis [61,62]. In vitro studies in human intestinal epithelial cells further demonstrate that these effects involve epithelial stress and apoptosis and may be partially mediated through reactive oxygen species (ROS) and nitric oxide (NO) signalling [63,64]. Poligeenan, a degraded derivative of carrageenan used in some industrial processes, exhibits even greater pro-inflammatory potential, inducing macrophage infiltration and epithelial ulceration in rodent models [65].

These findings collectively suggest that some UPF additives can mimic mechanisms observed in IBS, where low-grade mucosal inflammation, immune cell activation and impaired barrier function are hallmarks of disease. Moreover, human studies have reported elevated systemic inflammatory markers (e.g., IL-6, CRP, TNF-α) among individuals with higher UPF intake, supporting the notion that chronic exposure to these compounds may contribute to mucosal immune activation beyond the gut [66]. This low-grade inflammation could sensitize enteric nerves and amplify visceral hypersensitivity, a key driver of IBS symptoms [67].

### 9.3. Systemic and Metabolic Responses

Beyond the local intestinal environment, UPFs may promote systemic oxidative stress and metabolic dysregulation. Diets high in UPFs are typically rich in refined carbohydrates, saturated fats, and additives capable of generating reactive oxygen species (ROS) during digestion and absorption. Observational studies indicate that high UPF consumption correlates with increased plasma malondialdehyde levels, decreased activity of antioxidant enzymes (superoxide dismutase, glutathione peroxidase) and higher circulating DNA oxidation products [68,69].

Excessive oxidative stress may exacerbate epithelial damage and promote cytokine release, perpetuating gut inflammation. Additionally, postprandial glycaemic fluctuations and elevated lipid load from UPF-rich diets can disturb enteroendocrine hormone release (GLP-1, PYY, ghrelin), alter gut motility and modulate brain–gut signalling pathways involved in visceral sensitivity and mood regulation [7].

### 9.4. Translational Considerations

Although these mechanisms provide biological plausibility, it is important to distinguish between evidence derived from human versus preclinical studies. Findings on microbiota diversity, SCFA profiles and systemic oxidative stress come primarily from human cohorts, whereas detailed mechanistic insights—such as TLR4-NF-κB activation or specific additive-induced permeability changes—are mainly derived from animal and in vitro models. While translation to human physiology requires caution, these data collectively suggest that chronic consumption of UPFs can influence gut–immune and metabolic homeostasis in ways consistent with observed pathophysiological features of IBS.

## 10. Epidemiological Evidence Linking UPF to IBD

Increasing evidence points towards UPF consumption as a risk factor for inflammatory bowel disease (IBD), and meta-analyses investigating this association have been showing consistent results in recent years. A systematic review and meta-analysis by Narula et al., including five studies and a total of 1,068,425 participants, found an increased risk of developing Crohn’s disease (CD) in participants with higher consumption of UPF compared with those with lower UPF consumption (HR, 1.71; 95% CI, 1.37–2.14) [70]. Also, they found a lower risk of CD in participants with higher consumption of unprocessed or minimally processed foods compared with those with lower consumption of such foods (HR, 0.71; 95% CI, 0.53–0.94). Of note, no such associations were observed for ulcerative colitis (UC) [70]. These results are supported by a systematic review and dose–response meta-analysis by Babaei et al. from 2024, including 4,035,694 participants, which found that high intake of UPF was significantly associated with increased risk of IBD (RR 1.13; 95% CI, 1.06–1.21; *p* = 0.001), in particular for CD (RR 1.19; 95% CI, 1.00–1.41) [71].

As discussed above, several common additives are suggested to contribute to inflammation in IBD, such as modified starches, carrageenan, CMC and polysorbate 80. Interestingly, enteral nutrition formulas used in the treatment of active IBD are normally high in these additives. An analysis of 61 exclusive enteral nutrition (EEN) formulas used in the treatment of CD found that remission rates did not differ between EEN formulas with and without those food additives, a finding that challenges the perception that these substances may be harmful in IBD [72].

Insights from IBD pathophysiology may help illuminate mechanisms underlying IBS, due to considerable overlap in underlying mechanisms [73]. Many IBD patients report persisting IBS-like symptoms, despite remission of active inflammatory response. Although a formal IBS diagnosis is not made in this group, the prevalence of “IBS in IBD” surpasses the global prevalence of IBS by five-fold [74]. IBS symptoms in IBD patients in remission are suggested to share pathophysiological mechanisms with IBS, including gut microbial disturbances, increased intestinal permeability, visceral hypersensitivity, environmental triggers and involvement of gut–brain interaction. It has been questioned whether this condition is in fact a more subtle level of IBD activity that is unrecognized by available laboratory tests, which again questions the fact that IBS might be a subclinical form of IBD [75].

Although there is a lack of evidence from experimental studies on UPF intake in IBD patients, current clinical practice guidelines on diet for IBD advise restricting intake of UPF [76]. Taking the epidemiological evidence suggesting an association between UPF intake and IBD risk into consideration, along with the increasing evidence on the pro-inflammatory effects of additives and emulsifiers on IBD animal models, restricting UPF intake is arguably reasonable advice for IBD treatment, potentially transferable to IBS.

## 11. Clinical Implications

**Figure 2:**
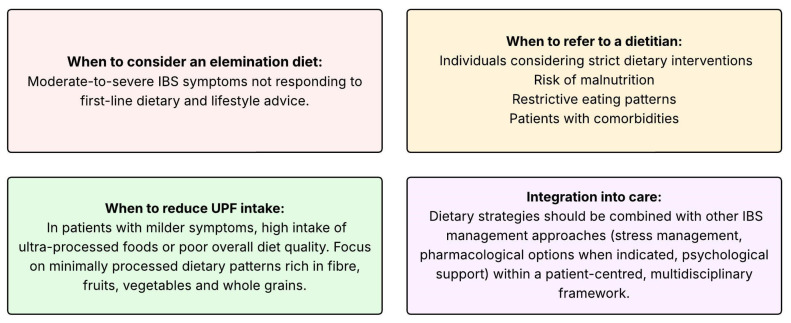
Clinical practice advice for dietary management of IBS.

Dietary modification remains a cornerstone in IBS management, and the accumulating evidence underscores the need for a balanced and individualized approach both to reduce the risk of and alleviate IBS symptoms. Clinical practice advice for dietary management of IBS is depicted in Figure 2. Clinicians should be aware that many patients’ self-initiate restrictive diets, which may alleviate symptoms but also increase the risk of nutritional inadequacy and unnecessary restrictive diets, can impair quality of life if applied without guidance. While the low-FODMAP diet still shows the strongest evidence for short-term symptom relief, emerging evidence highlights the importance of also evaluating dietary quality and not just focusing on eliminating triggers in IBS treatment [30]. Emerging data linking UPF consumption and Western-type dietary patterns to IBS and related disorders of gut–brain interaction suggest that focusing on dietary quality is clinically relevant. Encouraging a Mediterranean-style or otherwise diverse, minimally processed dietary pattern may provide both GI and extra-intestinal health benefits. In practice, clinicians should tailor dietary advice to individual symptom triggers, cultural food habits and psychosocial context, integrating dietary interventions into a multidisciplinary treatment plan.

## 12. Why Recommend Reduction of UPF in IBS?

Waiting for definitive, multi-arm UPF intervention trials to establish causality in IBS is neither realistic nor necessary for public health action. The exposure is combinatorially complex (thousands of products, matrices, and additives), patient responses are heterogeneous, and long-term randomized feeding trials that isolate every UPF dimension are infeasible and arguably unethical. Meanwhile, a convergent body of evidence—mechanistic (barrier/mucus disruption, altered fermentative ecology), short-term human feeding studies and prospective epidemiology—already satisfies several Bradford Hill–type considerations (plausibility, coherence, and consistency across designs). In such contexts, methodological pluralism and the precautionary principle justify risk-reducing action with ongoing evaluation. Crucially, UPF reduction is low-cost, reversible and carries broad co-benefits (diet quality, metabolic health), making the risk–risk trade-off favourable. We should therefore implement UPF-reduction strategies and clinical counselling prioritizing minimally processed foods, while embedding pragmatic trials and surveillance to refine who benefits most and why. This adaptive approach mitigates IBS burden now, without foreclosing stronger causal tests as the science advances.

## 13. Research Gaps and Limitations

Despite growing evidence linking dietary factors to risk for IBS, several important knowledge gaps remain, and the evidence on the effects of UPF on IBS to date holds several limitations. Available studies are limited, observational and rely on self-reported dietary data, limiting causal inference and raising the possibility of reporting bias. Furthermore, few prospective cohort studies have specifically examined the impact of habitual dietary patterns, diet quality and UPF consumption on incident IBS, and mechanistic pathways linking diet, the gut microbiome and IBS symptom generation require clarification.

## 14. Future Perspectives

Future research should prioritize large-scale, longitudinal cohorts with repeated dietary assessments, as well as pragmatic randomized trials comparing minimally processed diets and UPF diets meeting national dietary recommendations in IBS populations. Studies incorporating biomarkers, microbiome profiling and patient-centred outcomes, including quality of life and psychological well-being, will be critical to developing personalized, sustainable dietary recommendations for IBS.

## 15. Conclusions

Current evidence supports a possible link between UPF consumption and IBS. Increasing overall dietary quality and reducing UPF intake are promising complementary strategies to established dietary interventions. Future prospective studies and randomized controlled trials are needed to clarify the role of UPF in IBS pathogenesis and management.

## Figures and Tables

**Table 1:** Epidemiological studies reporting on the association between intake of ultra-processed foods and risk of irritable bowel syndrome (IBS).

Study/Cohort	Design	Participants (*n*)	UPF Assessment (Classification)	Dietary Assessment Method	IBS Incidence/ Prevalence in Study Population	Key Results for IBS
NutriNet-Santé (France, 2018) [48]	Cross-sectional	33,343 (76.4% woman, mean age 50.4)	% UPF by total food weight (NOVA)	Multiple repeated 24 h dietary records (web-based, ≥3 per participant)	3516 IBS cases (10.5% prevalence, Rome III criteria)	OR Q4 vs. Q1 ≈ 1.25 (95% CI: 1.12–1.39)
UK Biobank (UK, 2024) [47]	Prospective cohort	178,711 (53.1% female, mean age 55.8)	% of UPF in total diet as grams per day (NOVA)	Baseline touchscreen FFQ (≈200 items), linked to nutrient databases	2690 incident IBS cases over 11.3 years follow-up (~1.5% cumulative incidence)	HR ≈ 1.08 per 10% increase; HR Q4 vs. Q1 ≈ 1.19 (95% CI: 1.07–1.33)
ISFUN (Iran, 2025) [49]	Cross-sectional	1892 (54.5 female, mean age 39.6)	% of UPF in total diet as grams per day (NOVA)	Semi-quantitative validated Food Frequency Questionnaire (FFQ, 106 items)	100 IBS cases (5.3% prevalence, Rome IV criteria)	OR T1 vs. T3 for IBS ≈ 1.89 (95% CI: 1.01–3.55); strongest association observed among women

## References

[1] Lacy B.E., Pimentel M., Brenner D.M., Chey W.D., Keefer L., Long M.D., Ford A.C. (2021). ACG Clinical Guideline: Management of Irritable Bowel Syndrome. Am. J. Gastroenterol..

[2] Mearin F., Lacy B.E., Chang L., Chey W.D., Lembo A.J., Simren M., Spiller R.C. (2016). Bowel Disorders. Gastroenterology.

[3] Holtmann G.J., Ford A.C., Talley N.J. (2016). Pathophysiology of irritable bowel syndrome. Lancet Gastroenterol. Hepatol..

[4] Simrén M., Axelsson J., Gillberg R., Abrahamsson H., Svensson M., Ohlsson B. (2001). Food-related gastrointestinal symptoms in the irritable bowel syndrome. Digestion.

[5] Böhn L., Störsrud S., Törnblom H., Bengtsson U., Simrén M. (2013). Self-reported food-related gastrointestinal symptoms in IBS are common and associated with more severe symptoms and reduced quality of life. Am. J. Gastroenterol..

[6] Capili B., Anastasi J.K., Chang M. (2016). Addressing the Role of Food in Irritable Bowel Syndrome Symptom Management. J. Nurse Pract..

[7] Whelan K., Martin L., Staudacher H.M., Rossi M., Gibiino G., Bergeron K.F., Espin J., Taye B., Sokol H. (2024). Ultra-processed foods and food additives in gut health and disease. Nat. Rev. Gastroenterol. Hepatol..

[8] Lane M.M., Davis J.A., Beattie S., Gómez-Donoso C., Barnes T., Urban L., Buil J., Fearnley E., Baker P., Hall W.L. (2024). Ultra-processed food exposure and adverse health outcomes: Umbrella review of epidemiological meta-analyses. BMJ.

[9] Visioli F., Deiana M., Poli A., Galli C. (2023). The ultra-processed foods hypothesis: A product processed well beyond the basic ingredients in the package. Nutr. Res. Rev..

[10] Monteiro C.A., Cannon G., Lawrence M., Costa Louzada M.L., Pereira Machado P. (2019). Ultra-Processed Foods, Diet Quality, and Health Using the NOVA Classification System.

[11] Juul F., Martinez-Steele E., Parekh N., Monteiro C.A. (2025). The role of ultra-processed food in obesity. Nat. Rev. Endocrinol..

[12] Monteiro C.A., Moubarac J.C., Cannon G., Ng S.W., Popkin B. (2019). Ultra-processed foods: What they are and how to identify them. Public Health Nutr..

[13] Fardet A. (2016). Minimally processed foods are more satiating and less hyperglycemic than ultra-processed foods: A preliminary study with 98 ready-to-eat foods. Food Funct..

[14] Baker P., Machado P., Santos T., Sievert K., Backholer K., Hadjikakou M., Williams P., Woods J., Barquera S., Lawrence M. (2020). Ultra-processed foods and the nutrition transition: Global, regional and national trends, food systems transformations and political economy drivers. Obes. Rev..

[15] Gupta S., Stancliffe R.J., Seimon R.V., Halim M., Nowson C.A., Wark J.D., Gibbs R.A., Thomson R.L. (2021). Characterising percentage energy from ultra-processed foods by participant demographics, diet quality and diet cost: Findings from the Seattle Obesity Study (SOS) III. Br. J. Nutr..

[16] Moubarac J.C., Batal M., Louzada M.L.C., Martinez Steele E., Monteiro C.A. (2014). Processed and ultra-processed food products: Consumption trends in Canada from 1938 to 2011. Can J. Diet Pract. Res..

[17] Wang L., Liu J., Chen Y., Zhao Z., Wang Q. (2021). Trends in Consumption of Ultraprocessed Foods Among US Youths Aged 2–19 Years, 1999–2018. JAMA.

[18] Oka P., Parr H., Barberio B., Black C.J., Savarino E., Ford A.C. (2020). Global prevalence of irritable bowel syndrome according to Rome III or IV criteria: A systematic review and meta-analysis. Lancet Gastroenterol. Hepatol..

[19] El-Salhy M., Hatlebakk J.G., Gilja O.H., Hausken T. (2025). Quality of life, functional impairment and healthcare experiences of patients with irritable bowel syndrome in Norway: An online survey. BMC Gastroenterol..

[20] Canavan C., West J., Card T. (2014). Review article: The economic impact of the irritable bowel syndrome. Aliment. Pharmacol. Ther..

[21] Ford A.C., Lacy B.E., Talley N.J. (2020). Irritable bowel syndrome. Lancet.

[22] Camilleri M., Boeckxstaens G. (2023). Irritable bowel syndrome: Treatment based on pathophysiology and biomarkers. Gut.

[23] Berg L.K., Baines K.J., Krog J., Nordgaard-Lassen I., Hvas C.L., Mortensen E., Hansen J., Ankersen D.V., Sørensen S., Drewes A.M. (2020). Intestinal inflammatory profile shows increase in a diversity of biomarkers in irritable bowel syndrome. Scand. J. Gastroenterol..

[24] Güven E., Başpınar B., Atalay R. (2022). Relationship Between Systemic Immune-Inflammation Index and Irritable Bowel Syndrome. Turk. J. Gastroenterol..

[25] Fond G., Loundou A., Hamdani N., Boukouaci W., Dargel A., Oliveira J., Bengoufa D., Godin O., Brunel L., Boyer L. (2014). Anxiety and depression comorbidities in irritable bowel syndrome (IBS): A systematic review and meta-analysis. Eur. Arch. Psychiatry Clin. Neurosci..

[26] Bhattarai Y., Muniz Pedrogo D.A., Kashyap P.C. (2017). Irritable bowel syndrome: A gut microbiota-related disorder?. Am. J. Physiol. Gastrointest. Liver Physiol..

[27] Enck P., Mazurak N. (2018). Dysbiosis in Functional Bowel Disorders. Ann. Nutr. Metab..

[28] Casen C., Vebø H.C., Sekelja M., Hegge F.T., Karlsson F., Ciemniejewska E., Dzankic A., Friman V., Kristiansen K., Nielsen H.B. (2015). Deviations in human gut microbiota: A novel diagnostic test for determining dysbiosis in patients with IBS or IBD. Aliment. Pharmacol. Ther..

[29] Rodino-Janeiro B.K., Vicario M., Alonso-Cotoner C., Pascua-García R., Santos J. (2018). A Review of Microbiota and Irritable Bowel Syndrome: Future in Therapies. Adv. Ther..

[30] Dale H.F., Ling H., Helland L., Holte K., Rudi K., Midtvedt T., Færgestad T., Størvold G., Sævik K., Hov J.R. (2023). Diet-microbiota interaction in irritable bowel syndrome: Looking beyond the low-FODMAP approach. Scand. J. Gastroenterol..

[31] Lee J.Y., Kim H.S., Kim J., Park S.Y., Kim Y., Kim T.H. (2024). The human gut microbiome in health and disease: Time for a new chapter?. Infect. Immun..

[32] Hayes P.A., Fraher M.H., Quigley E.M. (2014). Irritable bowel syndrome: The role of food in pathogenesis and management. Gastroenterol. Hepatol. N. Y..

[33] Oświęcimska J., Słomka M., Bielicka A., Grzybowska-Chlebowczyk U. (2017). New insights into the pathogenesis and treatment of irritable bowel syndrome. Adv. Med. Sci..

[34] Cozma-Petruţ A., Nechita D., Sava I., Iorga M., Tănase F. (2017). Diet in irritable bowel syndrome: What to recommend, not what to forbid to patients!. World J. Gastroenterol..

[35] Nybacka S., Törnblom H., Bohn L., Simren M. (2024). Dietary Intake and Quality in Irritable Bowel Syndrome: A Comparative Study with Controls and the Association with Symptom Severity. Am. J. Gastroenterol..

[36] Eswaran S.L., Chey W.D., Han-Markey T., Ball S., Jackson K. (2016). A Randomized Controlled Trial Comparing the Low FODMAP Diet vs. Modified NICE Guidelines in US Adults with IBS-D. Am. J. Gastroenterol..

[37] Black C.J., Staudacher H.M., Ford A.C. (2022). Efficacy of a low FODMAP diet in irritable bowel syndrome: Systematic review and network meta-analysis. Gut.

[38] Wang J., Zhou Y., Zhang X., Chen Q., Yang H. (2021). A Low-FODMAP Diet Improves the Global Symptoms and Bowel Habits of Adult IBS Patients: A Systematic Review and Meta-Analysis. Front. Nutr..

[39] Singh P., Kumar R., Sharma A., Gupta N., Tiwari S., Aggarwal S. (2025). Efficacy of Mediterranean Diet vs. Low-FODMAP Diet in Patients with Nonconstipated Irritable Bowel Syndrome: A Pilot Randomized Controlled Trial. Neurogastroenterol. Motil..

[40] Kasti A.N., Tsiountouki K., Karageorgiou V., Panagiotakos D., Tsimihodimos V., Papanikolaou A. (2025). Clinical Trial: A Mediterranean Low-FODMAP Diet Alleviates Symptoms of Non-Constipation IBS-Randomized Controlled Study and Volatomics Analysis. Nutrients.

[41] Austin G.L., Dalton C., Hu Y., Khosla S., Rao S.S. (2009). A very low-carbohydrate diet improves symptoms and quality of life in diarrhea-predominant irritable bowel syndrome. Clin. Gastroenterol. Hepatol..

[42] Jaafari H., Houghton L.A., West R.M., Shuweihdi F., Staudacher H., Nikolova S., Ford A.C., Whorwell P.J., Bangdiwala S.I., Palsson O.S. (2025). Dietary Patterns Are Associated with Variations in the Global Prevalence and Severity of Rome IV Irritable Bowel Syndrome. Clin. Gastroenterol. Hepatol..

[43] Khayyatzadeh S.S., Nazem E., Mirmiran P., Azizi F. (2016). Dietary patterns and prevalence of irritable bowel syndrome in Iranian adults. Neurogastroenterol. Motil..

[44] Bolte L.A., Vich Vila A., Zhernakova A., Fu J., Wijmenga C., Franke L., Weersma R.K. (2021). Long-term dietary patterns are associated with pro-inflammatory and anti-inflammatory features of the gut microbiome. Gut.

[45] Taneri P.E., Kayser B., Kiefte-de Jong J.C., Glisic M., Fagherazzi G., Muka T. (2022). Association Between Ultra-Processed Food Intake and All-Cause Mortality: A Systematic Review and Meta-Analysis. Am. J. Epidemiol..

[46] Barbaresko J., Koch M., Schulze M.B., Nöthlings U. (2025). Ultra-processed food consumption and human health: An umbrella review of systematic reviews with meta-analyses. Crit. Rev. Food Sci. Nutr..

[47] Wu S., Li Y., Xu T., He L., Xie Q., Zhang X., Wang L. (2024). Ultra-Processed Food Consumption and Long-Term Risk of Irritable Bowel Syndrome: A Large-Scale Prospective Cohort Study. Clin. Gastroenterol. Hepatol..

[48] Schnabel L., Buscail C., Sabate J.M., Touvier M., Fezeu L., Druesne-Pecollo N., Deschasaux M., Latino-Martel P., Julia C., Kesse-Guyot E. (2018). Association Between Ultra-Processed Food Consumption and Functional Gastrointestinal Disorders: Results from the French NutriNet-Santé Cohort. Am. J. Gastroenterol..

[49] Haghighatdoost F., Farhadnejad H., Mirmiran P., Azizi F. (2025). The Association Between Ultra-Processed Foods Consumption and Disorders of Gut-Brain Interaction: The Isfahan Functional Disorders (ISFUN) Study. Neurogastroenterol. Motil..

[50] Juul F., Bere E. (2024). Ultra-processed foods—A scoping review for Nordic Nutrition Recommendations 2023. Food Nutr. Res..

[51] Hall K.D., Ayuketah A., Brychta R., Cai H., Cassimatis T., Chen K.Y., Chung S.T., Costa E., Courville A., Darcey V. (2019). Ultra-Processed Diets Cause Excess Calorie Intake and Weight Gain: An Inpatient Randomized Controlled Trial of Ad Libitum Food Intake. Cell Metab..

[52] Dicken S.J., McKenzie C.A., Jensen J.D., Nielsen S.M., Clausen J.S., Larsen M., Møller A., Beck A.M., Kristensen M. (2025). Ultraprocessed or minimally processed diets following healthy dietary guidelines on weight and cardiometabolic health: A randomized, crossover trial. Nat. Med..

[53] Preston J.M., Harrison A., Cohen P.A., Shapiro J., Hughes J., Smith L., Brown K., Taylor A., McDonald K. (2025). Effect of ultra-processed food consumption on male reproductive and metabolic health. Cell Metab..

[54] Mols R., Huybrechts I., Skeie G. (2025). High consumption of ultra-processed food and risk of colorectal cancer: The Norwegian Women and Cancer cohort study. Br. J. Nutr..

[55] Chassaing B., Miles-Brown J., Vijay-Kumar M., Gewirtz A.T., Wang L. (2022). Randomized Controlled-Feeding Study of Dietary Emulsifier Carboxymethylcellulose Reveals Detrimental Impacts on the Gut Microbiota and Metabolome. Gastroenterology.

[56] Zinöcker M.K., Lindseth I.A. (2018). The Western Diet–Microbiome-Host Interaction and Its Role in Metabolic Disease. Nutrients.

[57] Zangara M.T., Clark S.R., Parker W.J., Hale L.P., Lin Y., Wischmeyer P.E., Hsueh W., Iacomini J. (2022). Maltodextrin Consumption Impairs the Intestinal Mucus Barrier and Accelerates Colitis Through Direct Actions on the Epithelium. Front. Immunol..

[58] Karl J.P., Margolis L.M., Madslien E.H., Murphy N.E., Castellanos F., Aigner R., Eder K., McClung J.P. (2022). The Fecal Metabolome Links Diet Composition, Food Processing, and the Gut Microbiota to Gastrointestinal Health in a Randomized Trial of Adults Consuming a Processed Diet. J. Nutr..

[59] Atzeni A., Piras C., Perra A., Mura M., Pani A., Usai P., Contu P., Dore M.P. (2022). Association between ultra-processed food consumption and gut microbiota in senior subjects with overweight/obesity and metabolic syndrome. Front. Nutr..

[60] Bhattacharyya S., Ibrahim S.A., Smallwood M.A., Block S.R., Hecht G., Targan S.R., Rhodes J.M., Levine A. (2017). A randomized trial of the effects of the no-carrageenan diet on ulcerative colitis disease activity. Nutr. Healthy Aging.

[61] Wahnou H., Diallo A., Tchakoute H., Nguimfack A., Faye S., Nkoua F., Noubissi F., Ndomou S., Chouaib I., Tchinda R. (2025). Carrageenan and TLR4 Crosstalk: A Comprehensive Review of Inflammatory Responses in Animal Models. Recent. Adv. Inflamm. Allergy Drug Discov..

[62] Delahunty T., Recher L., Hollander D. (1987). Intestinal permeability changes in rodents: A possible mechanism for degraded carrageenan-induced colitis. Food Chem. Toxicol..

[63] Bhattacharyya S., Ibrahim S.A., Smallwood M.A., Block S.R., Hecht G., Targan S.R., Rhodes J.M., Levine A. (2008). Carrageenan induces cell cycle arrest in human intestinal epithelial cells in vitro. J. Nutr..

[64] Martino J.V., Van Limbergen J., Cahill L.E. (2017). The Role of Carrageenan and Carboxymethylcellulose in the Development of Intestinal Inflammation. Front. Pediatr..

[65] Benard C., Lau H., Forget G., Lambert M., Gosselin J. (2010). Degraded carrageenan causing colitis in rats induces TNF secretion and ICAM-1 upregulation in monocytes through NF-kappaB activation. PLoS ONE.

[66] Ciaffi J., Bertolotti M., Gazzaruso C., Barbieri F., Stefanelli A., Riso P., Del Bo’ C. (2025). Ultra-Processed Food Consumption and Systemic Inflammatory Biomarkers: A Scoping Review. Nutrients.

[67] Hasler W.L., Stanghellini V., Serra J., Pimentel M., Rao S.S., Camilleri M., Barbara G. (2022). Mast cell mediation of visceral sensation and permeability in irritable bowel syndrome. Neurogastroenterol. Motil..

[68] Quetglas-Llabrés M.M., Ortega R.M., Roca M., Vázquez M., Pérez-Fernández P., Hernáez Á., Pujol-Busquets G., Salas-Salvadó J. (2023). Oxidative Stress and Inflammatory Biomarkers Are Related to High Intake of Ultra-Processed Food in Old Adults with Metabolic Syndrome. Antioxidants.

[69] Haghighatdoost F., Farhadnejad H., Mirmiran P., Azizi F. (2023). Association between ultra-processed foods consumption and micronutrient intake and diet quality in Iranian adults: A multicentric study. Public Health Nutr..

[70] Narula N., Fedorak R.N., Rezaie A. (2023). Food Processing and Risk of Inflammatory Bowel Disease: A Systematic Review and Meta-Analysis. Clin. Gastroenterol. Hepatol..

[71] Babaei A., Sadeghi N., Esmaillzadeh A., Afshari M., Hajifaraji M., Akhlaghi M. (2024). The association of ultra-processed food consumption with adult inflammatory bowel disease risk: A systematic review and dose-response meta-analysis of 4,035,694 participants. Nutr. Rev..

[72] Logan M., Singh R., Tannock G., Guttman D.S., Rea M.C. (2020). Analysis of 61 exclusive enteral nutrition formulas used in the management of active Crohn’s disease-new insights into dietary disease triggers. Aliment. Pharmacol. Ther..

[73] Szałwińska P., Włodarczyk J., Spinelli A., Fichna J., Włodarczyk M. (2020). IBS-Symptoms in IBD Patients-Manifestation of Concomitant or Different Entities. J. Clin. Med..

[74] Wellens J., De Hertogh G., Ferrante M., Van Assche G., Vermeire S., Schrijvers R. (2025). Recent advances in clinical practice: Mastering the challenge-managing IBS symptoms in IBD. Gut.

[75] Tozlu M., Karadag O., Kiziltas S., Yildirim B., Alkim C., Sahin B. (2021). Dilemma in post-IBD patients with IBS-D symptoms: A 2020 overview. Expert Rev. Gastroenterol. Hepatol..

[76] Hashash J.G., Kane S.V., Regueiro M., Hanauer S.B., Katz J., Long M.D. (2024). AGA Clinical Practice Update on Diet and Nutritional Therapies in Patients with Inflammatory Bowel Disease: Expert Review. Gastroenterology.

